# CD147‐Conjugated Minocycline Nanomicelles Alleviate Neuroinflammation and Improve Neurological Function Following Intracerebral Hemorrhage

**DOI:** 10.1002/cns.71069

**Published:** 2026-08-20

**Authors:** Yang Liu, Yun Chen, Yuanyuan Liu, Zhe Li, Qingli Wang, V. Wee Yong, Ruixue Wei, Mengzhou Xue

**Affiliations:** ^1^ Department of Cerebrovascular Diseases The Second Affiliated Hospital of Zhengzhou University Zhengzhou Henan China; ^2^ Henan International Joint Laboratory of Intracerebral Hemorrhage and Brain Injury Zhengzhou University Zhengzhou Henan China; ^3^ Hotchkiss Brain Institute and Department of Clinical Neurosciences University of Calgary Calgary Alberta Canada

**Keywords:** anti‐CD147 antibody, brain targeting, intracerebral hemorrhage, minocycline, nanomicelles, neuroinflammation, neuroprotection

## Abstract

**Background:**

Intracerebral hemorrhage (ICH) remains one of the most devastating subtypes of stroke, characterized by high mortality and limited therapeutic options. Neuroinflammation is recognized as a critical determinant of secondary brain injury following ICH. Minocycline, a broad‐spectrum tetracycline antibiotic, has been shown to attenuate hematoma expansion, reduce blood–brain barrier (BBB) disruption, and improve neurological outcomes in preclinical ICH models. However, its clinical translation has been limited by poor aqueous solubility, low bioavailability, and dose‐related systemic toxicity.

**Methods:**

We designed a brain‐targeted nanotherapeutic system based on minocycline‐loaded polymeric nanomicelles conjugated with anti‐CD147 monoclonal antibodies (MINO@PNM@CD147). The nanomicelles were synthesized using a co‐solvent evaporation method, followed by covalent conjugation of CD147 antibodies via amide bond formation. The physicochemical properties, biocompatibility, and targeting efficiency of the formulation were characterized in vitro. Therapeutic efficacy was evaluated in a collagenase‐induced ICH mouse model using histopathological, biochemical, and behavioral assessments.

**Results:**

MINO@PNM@CD147 exhibited a uniform spherical morphology with an average hydrodynamic diameter of 13.5 nm and high colloidal stability. In vitro studies demonstrated excellent cellular compatibility and effective suppression of pro‐inflammatory mediators in lipopolysaccharide (LPS)‐activated BV‐2 cells. In vivo fluorescence imaging confirmed targeted accumulation of MINO@PNM@CD147 in perihematomal regions. Treatment markedly reduced neuronal degeneration, microglia and astrocyte activation, leukocyte infiltration, and cell apoptosis, while significantly attenuating the release of inflammatory mediators. Moreover, MINO@PNM@CD147 administration led to substantial improvements in neurological function compared with free minocycline or non‐targeted formulations.

**Conclusion:**

Our findings highlight that MINO@PNM@CD147 combines anti‐inflammatory efficacy with precise brain‐targeted delivery, thereby offering a potent and safe nanotherapeutic strategy for mitigating neuroinflammation and promoting recovery after ICH. This work provides a promising translational platform for nanomedicine‐based interventions in ICH.

## Graphical abstract

Schematic illustration of the design and therapeutic mechanism of MINO@PNM@CD147, which enables CD147‐mediated brain‐targeted delivery, suppresses neuroinflammation, alleviates neuronal injury, and enhances neurological recovery following intracerebral hemorrhage (ICH).

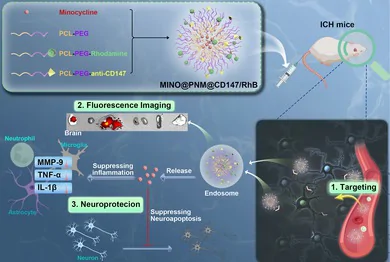

## 1 Introduction

Intracerebral hemorrhage (ICH) is a devastating subtype of stroke characterized by high mortality and long‐term disability rates [1, 2, 3]. The initial brain injury results from hematoma‐induced mechanical compression, followed by a multifactorial secondary brain injury that worsens the clinical outcomes [4, 5]. Among these secondary processes, neuroinflammation plays a central role in driving disease progression [2]. In particular, microglial activation, pro‐inflammatory cytokine release, and neuronal necrosis are key contributors to poor clinical outcomes following ICH [6]. Thus, targeting inflammation has emerged as a crucial strategy for improving prognosis in ICH patients [3]. Current clinical treatments for ICH primarily aim to manage symptoms, including hematoma evacuation and administration of recombinant activated factor VII to limit hematoma expansion [4, 7, 8]. However, these approaches mainly address the primary brain injury and have shown only modest benefits in clinical trials, underscoring the urgent need for more effective therapeutic strategies.

Minocycline, a broad‐spectrum tetracycline antibiotic, has demonstrated potent anti‐inflammatory, antioxidant, and matrix metalloproteinase (MMP)‐inhibitory properties, as well as the ability to reduce glutamate‐induced excitotoxicity [9, 10]. Our previous work and others have shown that minocycline suppresses the expression of extracellular matrix metalloproteinase inducer (EMMPRIN, CD147) and MMP‐9, mitigates blood–brain barrier (BBB) disruption, and improves neurological outcomes in acute ICH models [11]. Although intraperitoneal administration of minocycline has shown some therapeutic benefits [11, 12], its clinical application is limited by several pharmacokinetic drawbacks. Specifically, minocycline has poor water solubility and limited absorption, resulting in low systemic bioavailability. Moreover, the high doses (50–135 mg/kg) used in animal studies are associated with hepatotoxicity and nephrotoxicity [13]. Therefore, there is a great deal of interest in the use of effective minocycline for the improvement of its efficacy [14]. To overcome these limitations, nanomaterial‐based drug delivery systems have attracted increasing interest. Among these, self‐assembled polymeric nanomicelles, composed of amphiphilic block copolymers with hydrophilic shells and hydrophobic cores, offer a promising platform for encapsulating poorly soluble drugs [15]. The hydrophobic core facilitates drug loading and retention, while the hydrophilic shell enhances solubility and stability in physiological environments [16, 17]. For example, poly(ethylene glycol)‐block‐poly(ε‐caprolactone) (PEG‐PCL) has been widely employed in nanomedicine due to its biocompatibility, non‐toxicity, and degradability into metabolites that are either excreted or enter the tricarboxylic acid cycle [17]. This approach improves drug efficacy and reduces the potential side effects associated with systemic therapy.

**SCHEME 1:**
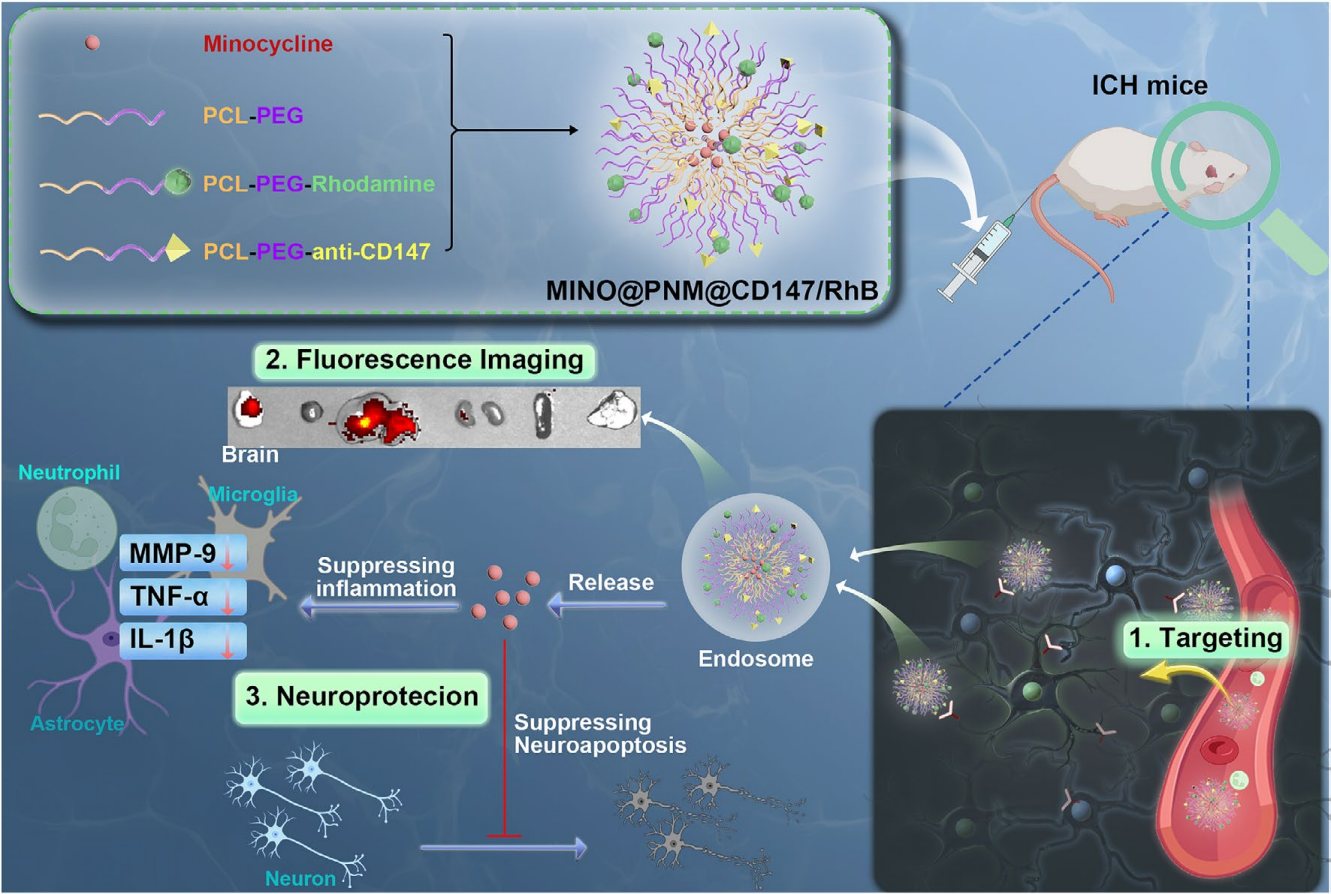
Schematic illustration of the design of MINO@PNM@CD147 and its therapeutic mechanism for alleviating neuroinflammation following intracerebral hemorrhage. MINO@PNM@CD147 enables CD147‐mediated brain‐targeted delivery, suppresses neuroinflammation, alleviates neuronal injury, and enhances neurological recovery following intracerebral hemorrhage.

Our previous studies found that CD147 is highly upregulated on activated microglia following ICH [18]. Targeting this molecule using a specific anti‐CD147 monoclonal antibody has shown therapeutic potential and safety in preclinical ICH models [18]. In the present study, as depicted in Scheme 1, we developed a minocycline‐loaded nanomicelle system (MINO@PNM) and further conjugated it with anti‐CD147 antibodies (MINO@PNM@CD147) to enable targeted delivery to inflamed brain regions. As illustrated in Scheme 1, we investigated the therapeutic efficacy of this brain‐targeted nanotherapeutic in vitro using lipopolysaccharide (LPS)‐stimulated BV2 microglial cells and in vivo using a collagenase‐induced ICH mouse model.

## 2 Materials and Methods

### 2.1 Preparation of MINO@PNM@CD147 Nanomicelles (NMs)

The minocycline‐loaded NMs were prepared by co‐solvent evaporation [19] and modified with antibody by amide reaction [20]. Briefly, 10 mg of minocycline and 200 μL trimethylamine were dissolved in 10 mL of methanol, and then the mixture was magnetically stirred at 200 rpm for 24 h in the dark. After the resulting solution was evaporated, 10 mL of tetrahydrofuran (THF) solution was added. PCL_3000_‐PEG_2000_ (7 mg), PCL_3000_‐Rhodamine B (RhB, 2 mg), and PCL_3000_‐PEG_2000_‐Maleimide (1 mg) were dissolved in the above THF mixture (2 mL); then, 10 mL of distilled water was added and stirred for 24 h at room temperature. After that, the THF was removed using a rotary evaporator to obtain solution A. Subsequently, 0.2 mg of CD147 (1 mg/mL) and cysteine (0.2 mg) were added to 500 μL of double‐distilled water to obtain solution B. *N*‐hydroxysuccinimide (NHS, 4 mg) and 1‐(3‐dimethylaMINOpropyl)‐3‐ethylcarbodiimide hydrochloride (EDC, 4 mg) were dissolved in dimethyl sulfoxide (DMSO, 4 mL) to obtain solution C. Solution D was obtained by mixing solution B and solution C for 48 h at room temperature. Finally, solution A and solution D were stirred for 24 h at room temperature and lyophilized to obtain MINO@PNM@CD147, which was stored at 4°C. All preparations were carried out under dark conditions.

### 2.2 Characterization of MINO@PNM@CD147 Nanomicelles

The hydrodynamic diameter and zeta‐potential of MINO@PNM@CD147 were measured by dynamic light scattering (DLS). The morphology of MINO@PNM@CD147 NMs was characterized using a transmission electron microscope (TEM) as described previously [21].

### 2.3 Characterization of Cytotoxicity

CCK‐8 assay was used to examine the effects of PCL_3000_‐PEG_2000_@PNM group, bulk minocycline group, and MINO@PNM@CD147 group on the cytotoxicity of cultured BV‐2 microglia. BV‐2 cells were cultured at 37°C in DMEM supplemented with 10% fetal bovine serum and 1% wt% penicillin/streptomycin in a 5% CO_2_ incubator. In brief, BV‐2 cells were plated in a 96‐well plate at density of 2 × 10^4^ cells/well and incubated for 24 h. The cultures were treated with different concentrations of PCL_3000_‐PEG_2000_–NPs (500, 250, 125, 62.5, 31.25, 15.63, 7.81, and 3.91 μg/mL), bulk minocycline and MINO@PNM@CD147‐RhB (50, 25, 12.5, 6.25, 3.13, 1.56, 0.78, and 0.39 μg/mL). After culturing for 24 h, we used the CCK‐8 kit to detect cytotoxicity. Each well was incubated with CCK‐8 reagent (10 μL) for 1 h, and the absorbance was measured at 450 nm.

### 2.4 In Vitro Cellular Uptake

BV‐2 cells were cultured in DMEM supplemented with 10% FBS and 1% penicillin/streptomycin at 37°C in a humidified incubator containing 5% CO_2_. The cells were seeded onto slide‐bottomed 24‐well plates at a density of 5 × 10^4^ cells per well in 0.5 mL of complete medium. After 24 h of culture, MINO@PNM@CD147 was added to each well at a MINO‐equivalent concentration of 1.6 μg/mL, which was selected based on the CCK‐8 cytotoxicity results to minimize cytotoxicity‐related interference. The cells were incubated for 2, 4, or 24 h to evaluate time‐dependent cellular uptake. At each indicated time point, the culture medium was removed, and the cells were washed three times with cold PBS (pH 7.4) to remove extracellular nanomicelles. The cells were then fixed with 4% paraformaldehyde (PFA) for 20 min at room temperature and rinsed three times with cold PBS. The slides were detached from the bottom of the 24‐well plates and mounted with antifade mounting medium containing 4′,6′‐diamidino‐2‐phenylindole (DAPI). The intracellular localization of MINO@PNM@CD147 in BV‐2 microglia was observed by fluorescence microscopy using excitation at 594 nm.

### 2.5 Quantitative Reverse Transcription‐Polymerase Chain Reaction (RT‐qPCR)

BV‐2 cells were seeded in 6‐well culture plates at a concentration of 2 × 10^6^ cells per well. The cells were cultured until they reached approximately 80% confluence, at which point the experiment was initiated. Subsequently, the cells were treated with 100 μg/mL LPS for 4 h. Afterward, free MINO (1.6 μg/mL) and MINO@PNM@CD147 (1.6 μg/mL for MINO) were added to the cells. The control group remained untreated, while the model group (LPS group) was treated with an equal volume of complete medium containing 100 ng/mL LPS. Total RNA was extracted following the instructions provided by the BioFlux Total RNA Extraction Kit, and subsequent experiments were conducted.

### 2.6 Experimental Animals

All animal experiments were approved by the Institutional Animal Care and Use Committee (IACUC) in accordance with the guidelines of the China Council on Animal Care. For the study, 8‐ to 10‐week‐old male C57BL/6 mice, weighing 20–23 g each, were provided by the Beijing Vital River Experimental Animal Centre (Beijing, China). The animals were allowed free access to food and water and were housed in designated specific pathogen‐free (SPF) rooms with controlled environmental conditions, including a temperature of 25°C, relative humidity (RH) of 55%, and a 12‐h light/dark cycle.

### 2.7 Generation of ICH Mouse Model

The animal study protocol was approved by the Institutional Animal Care and Use Committee. The ICH model was established as previously described [11]. Briefly, the mice were anesthetized with pentobarbital (40 mg/kg) intraperitoneally. Then animals were positioned on the brain stereotactic apparatus (RWD, Shenzhen, China). A total of 0.075 U of collagenase type VII (Sigma‐Aldrich, Milwaukee, WI, USA) was dissolved in 0.5 μL of sterile saline and injected into the mouse brain according to widely accepted coordinates (located in the right basal ganglia, 2.0 mm away from the midline, 3.7 mm under the skull, 3.5 mm from the bone surface). After the injection, the needle was held in the same position for 10 min to prevent reflux before the microsyringe needle was slowly withdrawn. At the end of the surgery, the burr hole was covered with bone wax, followed by wound suturing and antibiotic treatment. Then, the animals were placed back in their home cage.

### 2.8 Fluorescent Imaging of Nanomicelles

Mice with successful ICH surgery were prepared and randomly divided into two groups: MINO@PNM group and MINO@PNM@CD147 group. The RhB‐loaded nanomicelles (3 mg/kg minocycline) were injected through the tail vein at 2 h after the surgery. Mice were sacrificed at different time points (1 h, 2 h, 4 h, 1 day, and 7 day). The brain, heart, lung, liver, and kidneys were isolated using an IVIS (Xenogen) with an excitation wavelength of 535 nm and emission wavelength of 600 nm for RhB or RhB‐loaded nanomicelles.

### 2.9 Experimental Groups and Drug Treatment

For experimental treatment, mice were randomly distributed into ICH, ICH + Minocycline (MINO), ICH + MINO@PNM, ICH + MINO@PNM@CD147 (*n* = 4/group). Mice received MINO (3 mg/kg) or MINO‐loaded nanomicelles (3 mg/kg) by tail intravenous injection 2 h after ICH and once per day for 3 day post‐ICH induction, with equivalent volumes of saline being administered to mice in the other treatment groups.

### 2.10 Neurological Function Tests

Corner turn test: Experimental mice were placed in a 30‐degree corner of the arena. Then they were given a choice to turn either left or right, and the direction was recorded. The experiment was repeated 10 times for each animal, and the percentage of right turns was calculated. The right‐turn rate of the injured mice was higher than that of the control mice (~50%) [22].

Modified neurological severity score (mNSS): mNSS was examined at various time points after ICH to assess overall neurological deficits. The scale comprises five parts: motion, sensory, balance, reflex, and abnormal movements. The mNSS test was graded from 0 (normal performance) to 18 (maximum defect). Higher scores represent more severe neurological dysfunction [11].

### 2.11 Hematoma Volume Measurement

Following behavioral testing, the mice were euthanized. The animals were then perfused with PBS followed by 4% phosphate‐buffered PFA, and the brains were immediately removed. The fixed brains were trimmed from the rostral to the caudal portion of the damaged areas to prepare coronal sections with a thickness of 1 mm. These sections were sequentially arranged, and the ICH hematoma volume (in cubic millimeters) was evaluated using Image‐Pro Plus 5.0 image processing software. The hematoma volume was calculated using the previously reported formula: *V* = *t* × (*A*_1_ + *A*_2_ + ⋯ + *A*_*n*_), where *V* represents the hematoma volume, *t* is the section thickness, and *A* represents the area of the hematoma [18].

### 2.12 Paraffin‐Embedded Brain Tissue

#### 2.12.1 Preparation

Following euthanasia by administration of an overdose of sodium pentobarbital, the mice were transcardially perfused with PBS, followed by perfusion with 4% PFA. The entire brain of each animal was then collected and immersed in 4% PFA for fixation. Following fixation, the brain samples were paraffin‐embedded and sectioned into 5 μm‐thick coronal sections for subsequent analysis.

### 2.13 Hematoxylin and Eosin (H&E) Staining

The effect of MINO@PNM@CD147 treatment on neuronal loss in the perihematomal region of mice in an ICH model was assessed by H&E staining. Briefly, brain tissues were sectioned into 5 μm‐thick paraffin‐embedded slices, which were then dried at 50°C for at least 30 min. The sections were subsequently treated following the previously described protocol [11].

### 2.14 Fluoro‐Jade B (FJB) Staining

Fluoro‐Jade B (FJB) staining was performed to identify degenerating neurons in perihematomal regions following ICH. Briefly, brain tissues were sectioned into 5 μm thick paraffin‐embedded slices. For staining, sections were immersed sequentially in 100% ethanol for 3 min, 70% ethanol for 1 min, and distilled water for 1 min, followed by incubation in 0.06% potassium permanganate (KMnO_4_) for 15 min with gentle agitation to reduce background fluorescence. After rinsing with distilled water for 2 min, the slides were incubated in 0.0004% FJB solution (prepared by diluting 0.01% stock FJB in 0.1% acetic acid) for 30 min at room temperature in the dark. The sections were then rinsed three times with distilled water (1 min each), air‐dried on a 37°C heating plate for 20 min, cleared in xylene for 3 min, and coverslipped using neutral resin mounting medium.

### 2.15 Nissl Staining

Nissl staining was used to observe neuronal morphologic changes in the perihematomal areas 3 day after ICH, according to the manufacturer's protocol (#G1430, Solarbio, China). Images were obtained using an OLYMPUS microscope (BX53‐P, Olympus Co., Japan). For quantification of Nissl‐positive cells, four photographs with a field of view of 200× were randomly taken around the hematoma from each brain slice. These analyses were conducted by a researcher who did not know the experimental conditions.

### 2.16 TUNEL Staining

TUNEL staining was performed to evaluate cell death, including both necrosis and apoptosis, 3 day following ICH, according to the manufacturer's protocol (Vazyme Biotech, Nanjing, China). Mouse brains were collected and processed for tissue preparation as described previously. For the quantification of TUNEL‐positive cells, four images at 200× magnification were randomly captured around the hematoma from each brain slice. These analyses were conducted by a researcher who was unaware of the experimental conditions.

### 2.17 Immunofluorescence Staining

Mouse brains were cut into 5 μm thick coronal sections for immunofluorescence (IF) staining, as described previously [11]. The samples were incubated with Iba‐1 polyclonal antibody (microglia marker, 1:300, Wako, Japan), GFAP polyclonal antibody (astrocyte marker, 1:500, Abcam, Cambridge, MA, USA), MMP‐9 polyclonal antibody (1:100, Abcam, Cambridge, MA, USA), and MPO polyclonal antibody (1:300, Abcam, Cambridge, MA, USA) were incubated with brain tissue sections at 4°C overnight. Then, the sections were rinsed three times with PBS (pH = 7.4) thrice, followed by incubation with the corresponding fluorescent secondary antibody (Alexa Fluor 488‐conjugated goat anti‐rabbit) (Abcam, Cambridge, MA, USA) for 1 h in the dark at room temperature. Finally, the stained sections were mounted on glass slides using mounting medium with DAPI (Solarbio, Beijing, China). Fluorescent images were obtained using an OLYMPUS microscope (BX53‐P, Olympus Co., Japan). For positive cell counting, four microscopic fields in each brain section and three sections per mouse were examined.

### 2.18 Statistical Analysis

All data are presented as mean ± standard deviation (SD). Blinding assessment was applied in all analyses. One‐way analysis of variance (ANOVA) followed by Tukey's multiple comparisons test was used to compare differences between multiple groups. A *p* < 0.05 was considered statistically significant. All statistical analyses were performed using GraphPad Prism 6.0.

## 3 Results

### 3.1 Characterization of MINO@PNM@CD147

**FIGURE 1:**
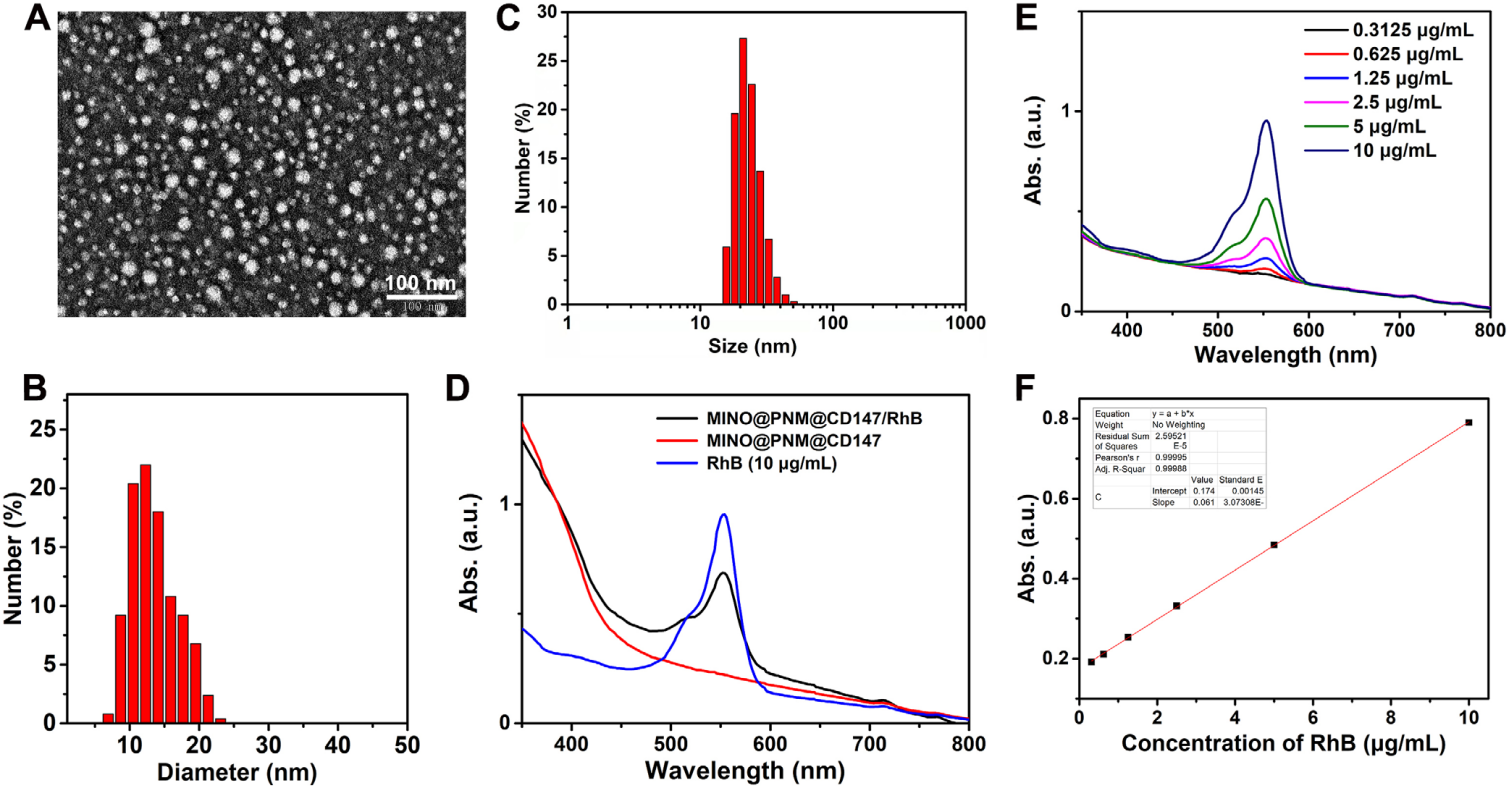
Characterization of MINO@PNM@CD147. (A) Transmission electron microscope (TEM) image of MINO@PNM@CD147. (B) diameter distribution of MINO@PNM@CD147 in Figure 1A. (C) DLS result of MINO@PNM@CD147. (D) UV–vis spectra of MINO@PNM@CD147/RhB, MINO@PNM@CD147, and RhB. (E) UV–vis absorption spectra of Rhodamine B (RhB) at different concentrations (0.3125, 0.625, 1.25, 2.5, 5, and 10 μg/mL). (F) The corresponding calibration curve plotting absorbance at 554 nm versus RhB concentration.

The preparation of MINO@PNM@CD147 was conducted via a co‐solvent evaporation process, following the methodology previously reported in the literature [18]. Transmission electron microscopy (TEM, Figure 1A,B) revealed that the obtained MINO@PNM@CD147 had an average diameter of approximately 13.5 nm, which was consistent with DLS analysis of 23.1 nm (Figure 1C). The changes occurring after RhB loading were detected using UV–vis spectroscopy. As illustrated in Figure 1D, the UV–vis spectra suggested the successful loading of RhB. The loading capacity was evaluated by UV–vis standard spectra of RhB (Figure 1E,F).

### 3.2 In Vitro Cell Behavior Study

**FIGURE 2:**
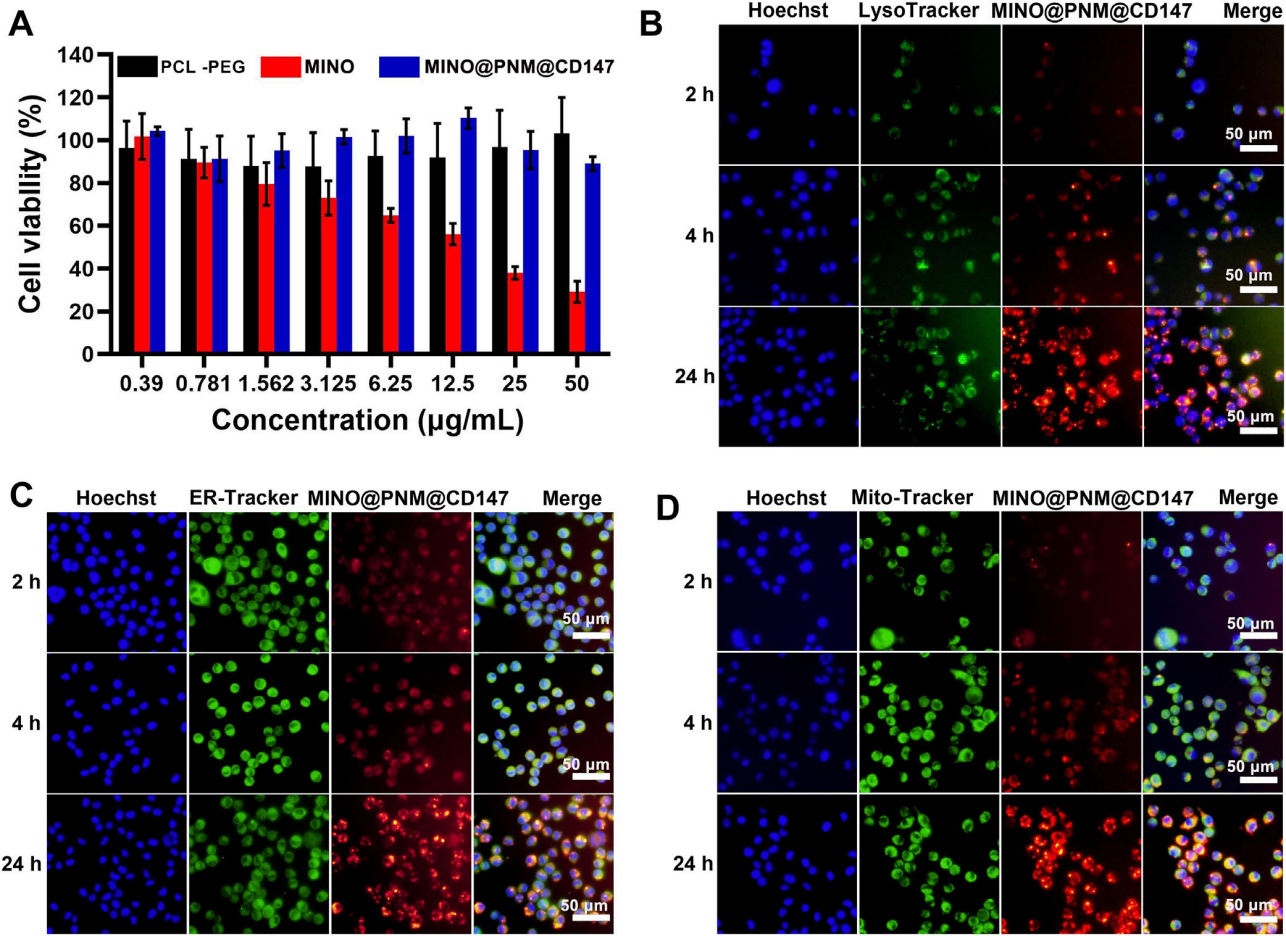
In vitro behavior study. (A) The cell viability of BV2 cells after incubation with MINO@PNM@CD147 for 24 h (*n* = 5/group). Confocal fluorescence images of BV2 cells after incubation with MINO@PNM@CD147/RhB in (B) lysosomes, (C) endoplasmic reticulum, and (D) mitochondria.

Next, we investigated the cellular behavior of MINO@PNM@CD147. Before assessing the cytotoxicity of MINO@PNM@CD147, we initially assessed the cytotoxicity of MINO and PEG‐PCL with Cell Counting Kit‐8 (CCK‐8) and showed that PCL‐PEG had no appreciable cytotoxicity after 24 h incubation (Figure 2A). However, after 24 h incubation, a notable decline in cellular viability was observed in MINO‐treated cells the viability of BV‐2 cells treated with MINO@PNM@CD147, was observed to be as high as 88% after 24 h of incubation. The results indicated that the nanomicelle enhanced the biocompatibility of MINO. To further evaluate neuronal safety, CCK‐8 assay was performed in HT‐22 cells. Free MINO reduced cell viability in a concentration‐dependent manner, whereas MINO@PNM@CD147 maintained high viability across the tested concentration range, indicating markedly improved neuronal biocompatibility and reduced neurotoxicity compared with free MINO (Figure S1). To gain further insight into the behavior of MINO@PNM@CD147 in cells, we attached the fluorescent dye rhodamine B (RhB) to the surface of MINO@PNM@CD147. Subsequently, fluorescence imaging of BV2 cells was conducted following treatment with MINO@PNM@CD147/RhB for different incubation periods (Figure 2B–D). The fluorescence images demonstrated that the fluorescence signal increased over time. The high degree of overlap of the MINO@PNM@CD147/RhB fluorescence signal and the LysoTracker signal indicated that MINO@PNM@CD147/RhB was successfully internalized by endocytosis.

### 3.3 MINO@PNM@CD147 Attenuated Neuroinflammation Induced by LPS In Vitro

**FIGURE 3:**
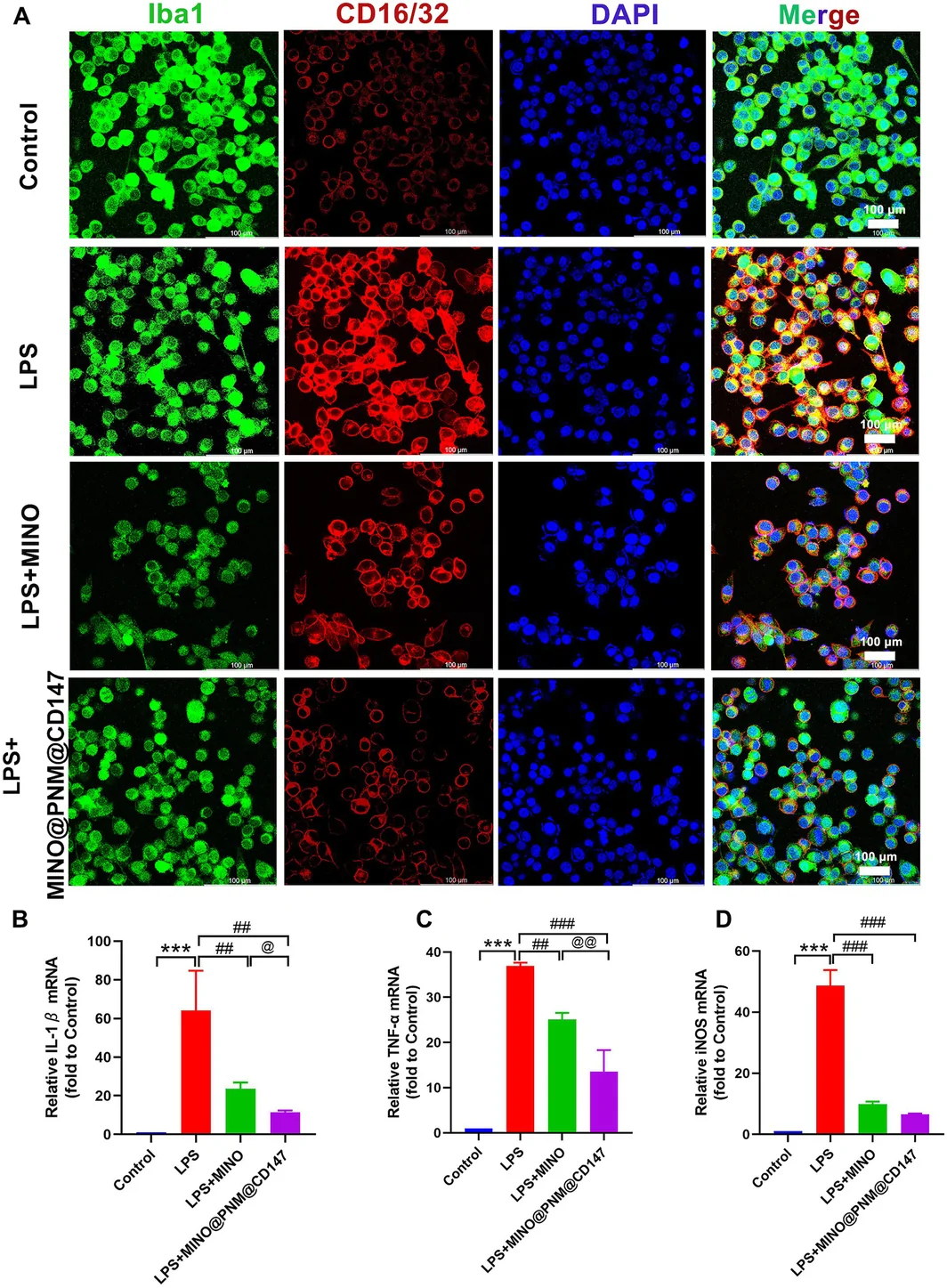
The anti‐inflammation effects of MINO@PNM@CD147 in vitro. (A) Representative images of double immunofluorescent analysis using Iba1 antibodies (green) and pro‐inflammatory marker CD16/32 (red) in BV2 cells stimulated with LPS for 4 h and then treated with different drugs for 24 h (*n* = 4). Scale bar = 100 μm. (B–D) Real‐time quantitative reverse transcription PCR (RT‐qPCR) was used to analyze the mRNA expression of IL‐1β (B), TNF‐α (C), and iNOS (D) in BV‐2 cells stimulated with LPS for 4 h and then treated with MINO@PNM@CD147 for 24 h. ***p < 0.001 compared with Control group, ^##^ *p* < 0.01 and ^###^ *p* < 0.001 compared with LPS group, ^@^ *p* < 0.05 and ^@@^ *p* < 0.01 compared with LPS + MINO group. (*n* = 4).

Considering that the progression of brain injury after ICH is accompanied by a strong inflammatory response, in which microglia play a pivotal role in the initiation and development of neuroinflammation [23, 24]. Accordingly, we proceeded to investigate the therapeutic effects of MINO@PNM@CD147 in vitro. We chose the classic LPS‐induced BV2 model of inflammation to simulate the inflammatory state in the brain after ICH [25]. First, we stimulated BV‐2 cells using LPS (100 ng/mL). Then, each experimental group was treated with MINO (1.6 μg/mL) and MINO@PNM@CD147 (1.6 μg/mL for MINO) for 24 h, respectively. As shown in Figure 3A, immunofluorescence images indicated that the fluorescent signal of the CD16/32 (M1 microglia marker) increased significantly in the LPS group, indicating that a pro‐inflammatory environment had been induced (Figure 3A). After a series of treatments, both free MINO and MINO@PNM@CD147 treatment reduced CD16/32 expression. To further evaluate the inhibitory effect of MINO@PNM@CD147 on the pro‐inflammatory activation of microglia, we examined the expression of key M1 microglia markers using real‐time PCR. As illustrated in Figure 3B–D, LPS stimulation resulted in a significant increase in the expression levels of TNF‐α, IL‐1β and INOS in BV‐2 cells. In contrast, MINO@PNM@CD147 markedly reduced the expression of these pro‐inflammatory markers.

### 3.4 Evaluation of the Therapeutic Efficacy of MINO@PNM@CD147 in ICH Mice

**FIGURE 4:**
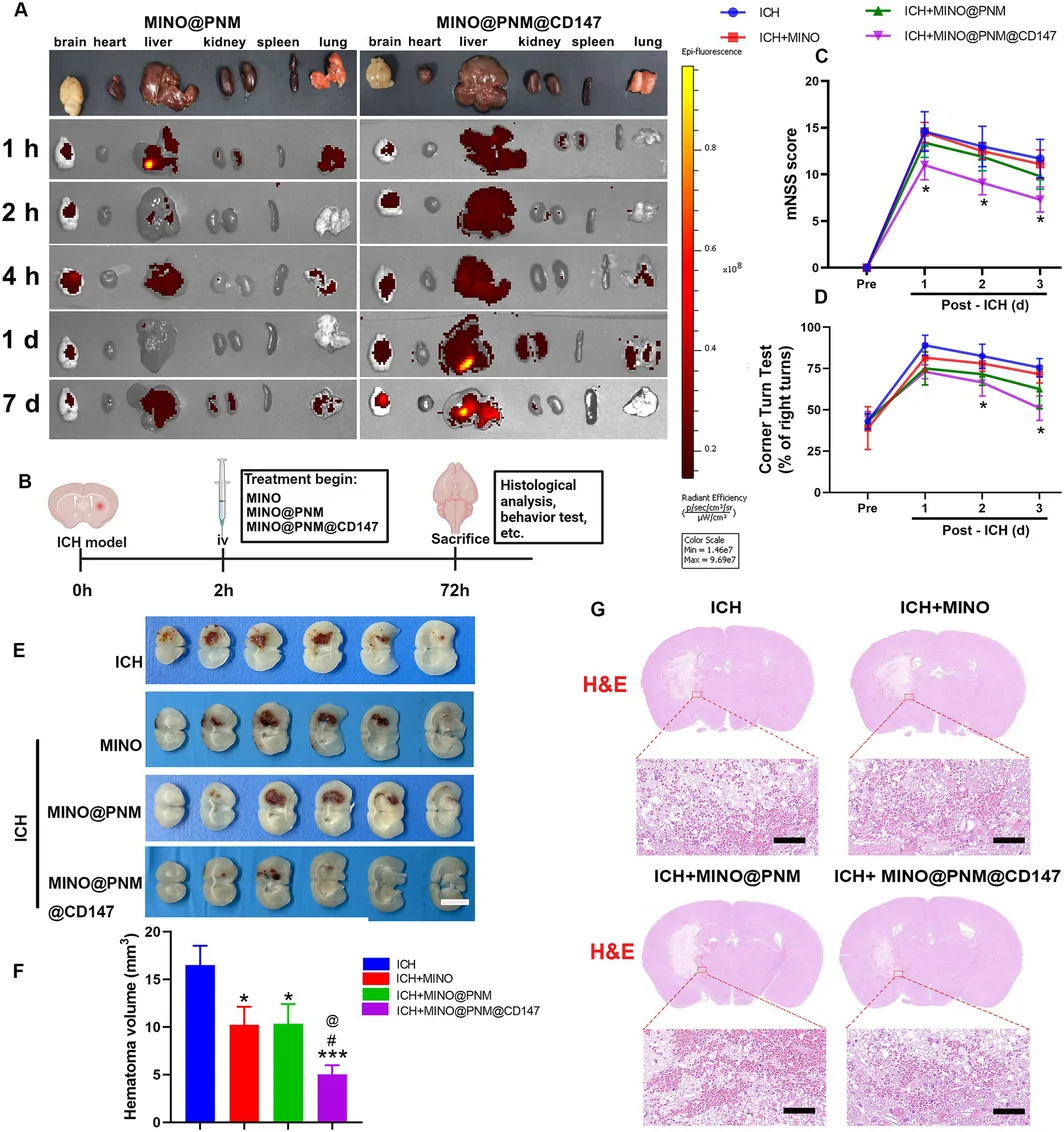
The therapeutic efficacy evaluation of MINO@PNM@CD147 in ICH mouse. (A) in vivo fluorescence imaging of organs was performed to study the distribution of drugs injected into the tail vein. Organs from left to right: Brain, heart, liver, kidney, spleen and lung. (B) The diagram above shows the protocol of the experiment. (C) Modified neurological severity score of mice on day(s) 1, 2, and 3 post‐ICH. (*n* = 10). (D) Corner turn test results of mice on day(s) 1, 2, and 3 post‐ICH. (*n* = 10 per group). (E) Images of hematomas in ICH mouse brain slices. (*n* = 3 per group), Scale bar = 0.5 cm. (F) Corresponding hematoma volume of different groups analyzed. (G) Representative photomicrographs of the H&E staining for the different treatments. (*n* = 4 per group). *p < 0.05 and ***p < 0.001compared with ICH, ^#^ *p* < 0.05 compared with ICH + MINO, ^@^ *p* < 0.05 compared with MINO@PNM.

To further evaluate the therapeutic efficacy of MINO@PNM@CD147 in ICH mice, we established the ICH mouse model to mimic clinical ICH. In addition, to confirm whether MINO@PNM@CD147 contributes to the brain injury, we examined the brain tissue distribution of RhB‐labeled MINO@PNM@CD147 in mice using a fluorescence imaging system. We employed C57BL/6 ICH mice to study the behavior of MINO@PNM@CD147/RhB in vivo. As the fluorescence of RhB overlaps with the autofluorescence of mice (mainly from fur and blood), ex vivo fluorescence imaging (Figure 4A) of mice was performed to further monitor RhB after intravenous injection of MINO@PNM@CD147/RhB (at a dose of 3 mg/kg minocycline). The fluorescent signals in the brain were enhanced 1 h after injection of both MINO@PNM/RhB and MINO@PNM@CD147/RhB. It is noteworthy that, following 1 day of injection, the fluorescence signal in the brains of mice that had been injected with MINO@PNM@CD147/RhB was significantly stronger than that of mice injected with MINO@PNM/RhB. The strong fluorescent signal in the brains of mice suggested that MINO@PNM@CD147/RhB accumulated in the brain and facilitated treatment of ICH.

We next evaluated the therapeutic effects of various MINO‐based formulations (Figure 4B). First, neurological function was assessed during the first 3 day post‐ICH using the mNSS and the corner turn test. Mice in the ICH group exhibited significantly elevated neurological deficit scores (Figure 4C,D). Treatment with either free MINO or nanomicelle‐based formulations improved neurological outcomes, with MINO@PNM@CD147 showing the most pronounced effect across multiple time points. Hematoma volume was measured on day 3 post‐ICH using coronal brain sections. As expected, the ICH group exhibited a large hematoma volume (Figure 4E,F). All MINO‐treated groups showed a significant reduction in hematoma size, with the greatest effect observed in the MINO@PNM@CD147‐treated mice. As shown in Figure 4G, in the collagenase‐induced ICH mouse model, the MINO@PNM@CD147‐treated group displayed a reduced lesion area and fewer signs of pathological damage around the hematoma. Collectively, these results demonstrate that the engineered MINO@PNM@CD147 nanomicelles effectively target the brain, alleviate neurological deficits, reduce hematoma volume, and mitigate secondary brain injury, highlighting their promise as a therapeutic strategy for ICH.

### 3.5 MINO@PNM@CD147 Reduced Cell Death After ICH

**FIGURE 5:**
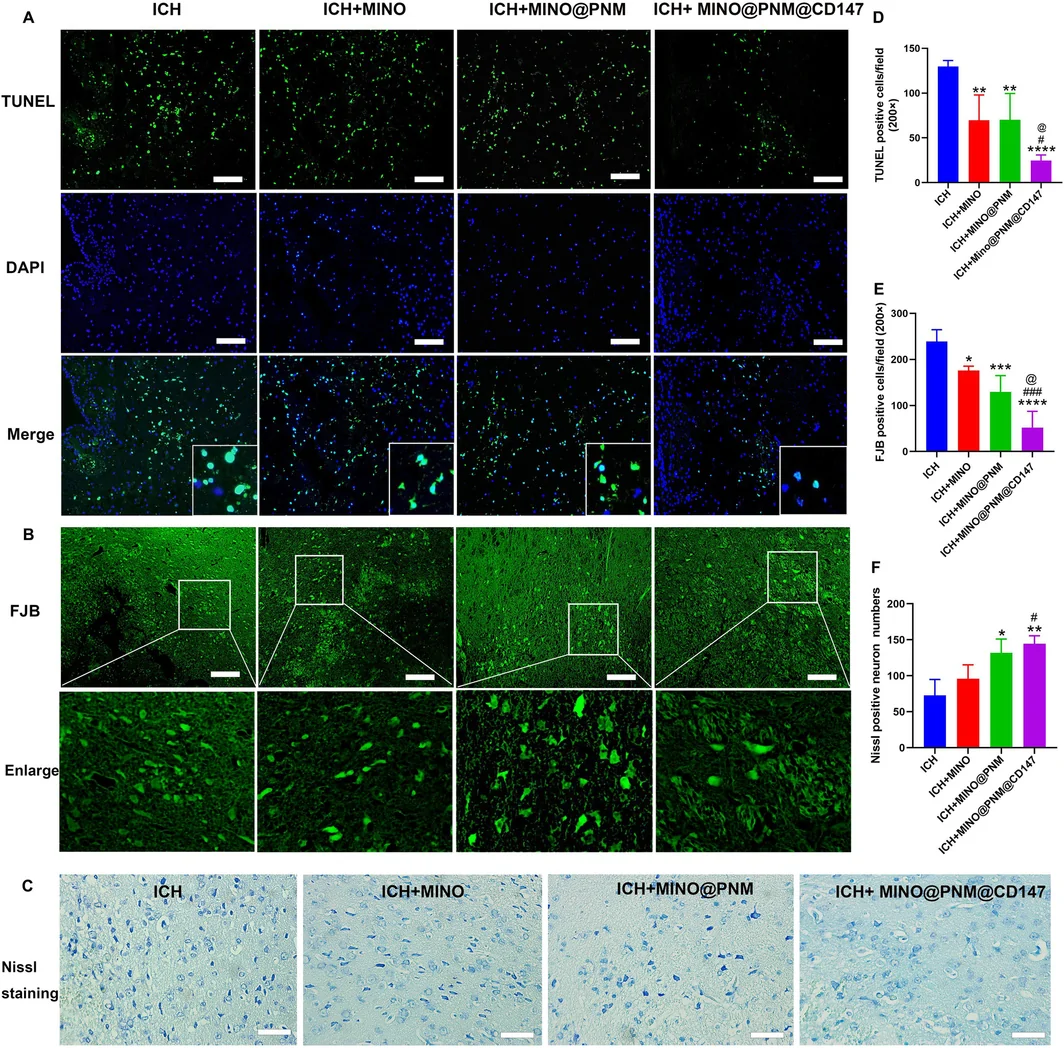
MINO@PNM@CD147 administration alleviated cell death in ICH mice. (A) Representative images of the TUNEL staining (green). (B) Representative images of the FJB staining (green). (C) Representative images of the Nissl staining of the perihematomal brain tissue. (D) Statistical analysis of the TUNEL positive cells in the different groups. (E) Statistical analysis of the FJB cells in the different groups. (F) Statistical analysis of the Nissl positive neurons in the different groups. *p < 0.05, **p < 0.01, ***p < 0.001 and ****p < 0.0001 compared with ICH; ^#^ *p* < 0.05 and ^###^ *p* < 0.001 compared with ICH + MINO; ^@^ *p* < 0.05 compared with MINO@PNM. (*n* = 4 per group). Scale bar = 100 μm.

The basal ganglia are a common site of primary ICH and play a crucial role as key relay stations for somatosensory and motor pathways [26]. After ICH, the survival of neurons in this region provides the structural basis for functional recovery in patients [27]. To assess the neuroprotective effects of MINO@PNM@CD147 in ICH mice, we conducted the following experiments. First, TUNEL staining was performed to determine apoptosis in the hemorrhagic region after different treatments. Compared with the ICH group, all treatment groups—MINO, MINO@PNM, and MINO@PNM@CD147—exhibited reduced numbers of TUNEL‐positive cells, with MINO@PNM@CD147 showing the greatest reduction (Figure 5A,D). Neuronal degeneration after ICH was performed by FJB staining. Both the MINO and MINO@PNM groups showed a marked reduction in FJB‐positive cells compared with the ICH group. Notably, the MINO@PNM@CD147 group demonstrated the most pronounced neuroprotection (Figure 5B,E). Meanwhile, Nissl staining was used to assess neuronal integrity in the perihematomal region (Figure 5C). As shown in Figure 5F, all treatment groups showed significantly reduced neuronal damage compared with the ICH group. Moreover, neuronal morphology appeared more regular and organized in the MINO@PNM@CD147 group, suggesting superior structural preservation. Collectively, these data indicate that the engineered nanomicelle formulation MINO@PNM@CD147 exerts robust neuroprotective effects following ICH.

### 3.6 MINO@PNM@CD147 Alleviated Neuroinflammation in ICH Mice

**FIGURE 6:**
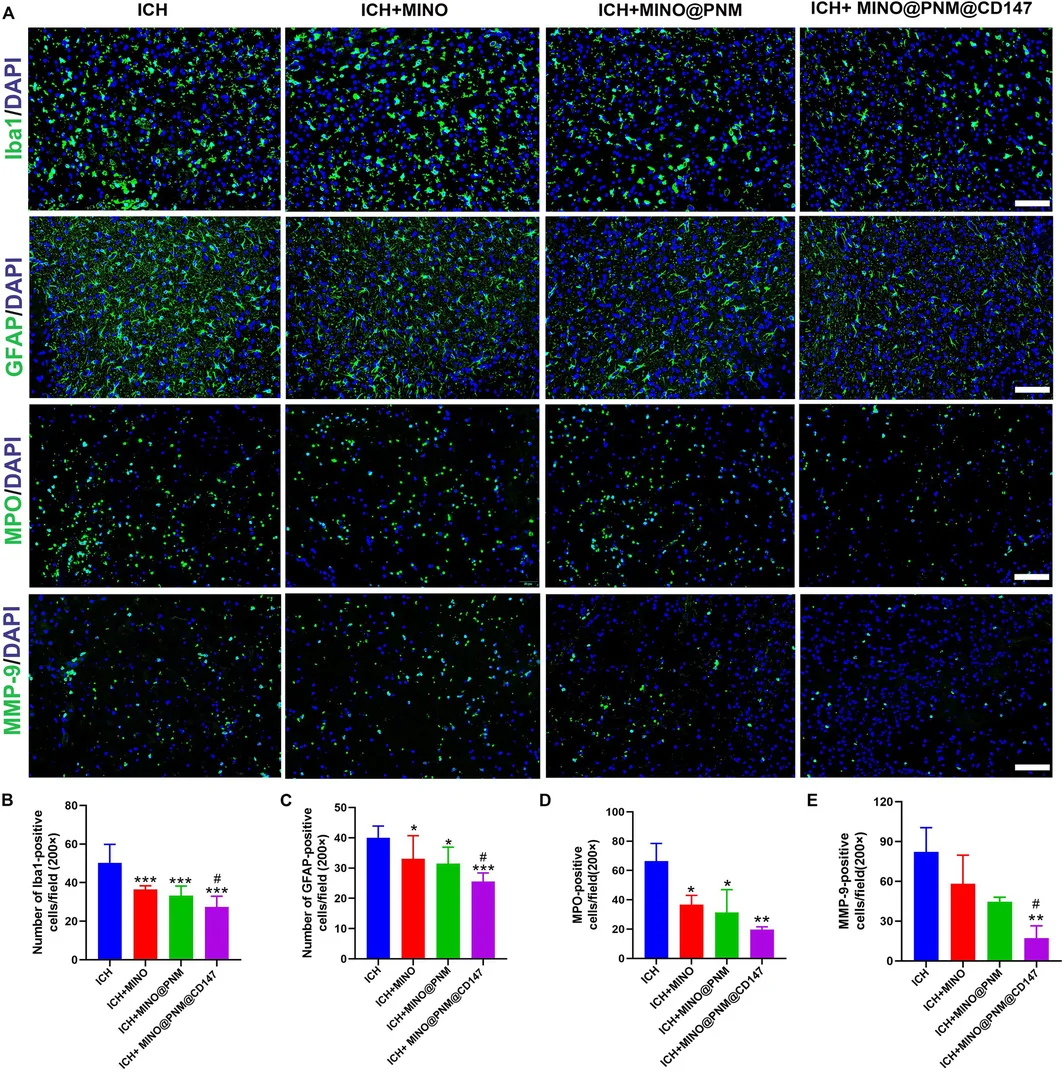
Therapeutic effects of MINO@PNM@CD147 suppression on inflammation response after ICH. (A) Representative images of Iba‐1, GFAP, MPO and MMP‐9 expression in brain sections of ICH mice from different treatment groups examined by immunofluorescent staining. (B) Statistical analysis of the Iba1‐positve cells in the perihematomal areas treated with different treatments. (C) Statistical analysis of the GFAP‐positive cells in the perihematomal areas treated with different treatments. (D) Statistical analysis of the MPO‐positive cells treated with different treatments. (E) Statistical analysis of the MMP‐9‐positve cells in the hematoma areas treated with different treatments. *p < 0.05, **p < 0.01 and ***p < 0.001 compared with ICH; ^#^ *p* < 0.05 and ^##^ *p* < 0.01 compared with ICH + MINO; ^@^ *p* < 0.05 compared with MINO@PNM. (*n* = 4 per group). Scale bar = 100 μm.

During ICH‐induced injury, extensive glial activation and neutrophil infiltration contribute to a pronounced inflammatory response and glial scar formation, ultimately exacerbating neuronal damage [2, 28]. Among these cell types, microglia and astrocytes are resident glial cells of the CNS, whereas neutrophils are peripheral immune cells that invade the brain. The activation of these cells reflects the degree of neuroinflammation. Anti‐inflammatory strategies are therefore considered promising therapeutic approaches for ICH [2]. To evaluate the anti‐inflammatory potential of MINO@PNM@CD147, we performed immunofluorescence staining to assess the expression levels of Iba‐1 and GFAP in the perihematomal region 3 day post‐ICH. As shown in Figure 6A,B, ICH significantly increased microglial activation, evidenced by elevated Iba‐1 expression and enlarged cell bodies. Treatment with MINO@PNM@CD147 significantly reduced Iba‐1 levels compared with free MINO and MINO@PNM. Similarly, reactive astrocytes exhibited hypertrophic morphology and increased GFAP expression following ICH. However, treatment with MINO@PNM@CD147 substantially reduced the area of GFAP‐positive astrocytes compared with the other treatment groups (Figure 6A,C). In addition to glial activation, ICH‐induced pathology also involves leukocyte infiltration, particularly neutrophils, which aggravate secondary brain injury. To investigate whether MINO@PNM@CD147 could mitigate neutrophil infiltration, we conducted immunofluorescence staining for myeloperoxidase (MPO), a neutrophil marker. As shown in Figure 6A,D, the number of MPO‐positive cells was markedly decreased in the MINO@PNM@CD147 group compared with the free MINO and MINO@PNM groups. MMP‐9, a key inflammatory mediator secreted by microglia, astrocytes, and infiltrating neutrophils, plays a critical role in ICH‐induced injury [29, 30]. Immunofluorescence analysis revealed that MINO@PNM@CD147 treatment significantly decreased MMP‐9‐positive cells in the perihematomal region (Figure 6A,E). These effects are likely attributable to the combined anti‐inflammatory properties of minocycline and the targeting capability of anti‐CD147.

### 3.7 Safety Evaluation of MINO@PNM@CD147 Nanomicelles

To assess the in vivo safety of the nanomicelles, major organs were harvested from mice and subjected to hematoxylin and eosin (H&E) staining. Histological analysis revealed no significant pathological abnormalities in the examined tissues, indicating that intravenous administration of MINO@PNM@CD147 nanomicelles did not induce observable organ toxicity (Figure S2). These results collectively suggest that the developed nanomicelle formulation exhibits a favorable safety profile in vivo.

## 4 Discussion

In this study, we successfully fabricated and characterized minocycline‐loaded polymeric nanomicelles conjugated with anti‐CD147 monoclonal antibodies (MINO@PNM@CD147) and demonstrated their therapeutic efficacy in a mouse model of ICH. Our results indicate that this targeted nanoplatform significantly attenuates neuroinflammation, mitigates secondary brain injury, and promotes neurological recovery after ICH.

ICH triggers a cascade of detrimental events, including neuronal apoptosis, BBB disruption, and an intense inflammatory response. Without timely intervention, excessive inflammation in the perihematomal region amplifies oxidative stress and exacerbates neuronal death, ultimately hindering neurological recovery [24, 31]. Therefore, early modulation of neuroinflammation is a key therapeutic strategy [32, 33], with microglial activation recognized as a central driver of secondary brain injury [6]. Activated microglia release pro‐inflammatory cytokines and reactive oxygen species (ROS), which intensify tissue damage and delay functional recovery [34].

Our previous studies identified CD147, a transmembrane glycoprotein markedly upregulated in activated microglia, as a promising therapeutic target for regulating neuroinflammatory cascades [11]. In this work, we designed MINO@PNM@CD147 to leverage CD147 as a molecular anchor for delivery of minocycline in the perihematomal area. Fluorescence imaging confirmed the preferential accumulation of MINO@PNM@CD147/RhB in the brain, suggesting successful BBB penetration and targeted drug delivery. This targeted strategy maximizes drug concentration at the lesion site while minimizing systemic exposure and off‐target effects.

Minocycline, a second‐generation tetracycline antibiotic, possesses robust anti‐inflammatory and neuroprotective properties across several CNS disorders, including ICH [11, 35, 36]. However, its clinical utility is hindered by poor water solubility, rapid systemic clearance, and the need for high or repeated dosing. Nanomicelle‐based delivery systems offer a compelling solution by improving solubility, pharmacokinetics, and tissue targeting of hydrophobic drugs. Our MINO@PNM@CD147 formulation enhanced the aqueous solubility and stability of minocycline, enabling efficient BBB penetration and accumulation in inflamed brain regions.

Importantly, the material innovation lies in the integration of multiple functionalities within a single nanoplatform: (1) the polymeric micelle core facilitates solubilization of hydrophobic minocycline, (2) the anti‐CD147 surface modification enables active targeting to inflamed microglia, and (3) the nanoscale structure ensures efficient brain accumulation with minimal systemic toxicity. This multifunctional integration confers superior therapeutic precision compared to conventional formulations.

CD147, initially studied as an oncological target [37, 38], has recently emerged as a novel therapeutic target in stroke and ICH [18, 39]. While several CD147‐targeting strategies have been explored in oncology, their application in neurological diseases remains limited [40]. Our previous work confirmed that functional blockade of CD147 using monoclonal antibodies ameliorates acute ICH pathology in mice [18]. Building upon this, the present study demonstrates that conjugation of anti‐CD147 antibodies to minocycline‐loaded micelles enhances site‐specific drug accumulation in areas of microglial activation. This targeted delivery significantly downregulated key inflammatory mediators such as TNF‐α and IL‐1β both in vitro, validating the efficacy of CD147‐guided delivery in neuroinflammatory contexts.

Overall, MINO@PNM@CD147 offers a promising therapeutic platform that addresses multiple challenges in ICH treatment. First, its site‐directed delivery system ensures therapeutic precision by localizing anti‐inflammatory effects at the epicenter of injury. Second, the nanocarrier system overcomes longstanding pharmacokinetic limitations of minocycline. Third, the incorporation of CD147‐targeting confers active specificity, reducing off‐target effects.

While this study provides robust evidence for the neuroprotective potential of our nanoplatform, certain limitations remain. The therapeutic intervention was initiated at 2 h post‐ICH, which serves as a critical proof‐of‐concept for early‐stage intervention. However, considering the delays often encountered in clinical diagnosis and patient transport, this early window may not fully capture the complexities of real‐world clinical scenarios. To further validate the translational potential of this system, future studies should evaluate its efficacy across more delayed therapeutic windows (e.g., 6 or 24 h after ICH) to better accommodate the pathological progression of stroke in human patients. Nevertheless, challenges remain. Optimization of target specificity is necessary to avoid unintended interactions with CD147‐expressing non‐microglial cells. Additionally, long‐term safety, immunogenicity, and biodegradability of the nanocarrier system warrant thorough investigation in preclinical models.

In conclusion, our findings establish MINO@PNM@CD147 as a novel, targeted nanotherapeutic system capable of effectively modulating neuroinflammation and protecting against ICH‐induced brain injury. This work provides a solid foundation for the future development of ligand‐guided nanotherapeutics for CNS diseases.

## 5 In Conclusions

In summary, minocycline‐loaded nanomicelles were successfully fabricated using a simple solvent evaporation method and subsequently functionalized with a CD147 monoclonal antibody. These nanomicelles were thoroughly characterized by TEM, DLS, and in vitro cytotoxicity assays. Their therapeutic potential was evaluated both in BV‐2 microglial cells in vitro and in a mouse model of ICH. The results demonstrated that the nanomicelles effectively reduced hematoma volume, alleviated neuronal degeneration, attenuated cell death, and suppressed inflammatory cell infiltration, ultimately promoting neurological recovery. Overall, CD147‐targeted nanomicelle delivery of minocycline represents a promising strategy to modulate neuroinflammation and enhance therapeutic efficacy in ICH treatment.

## Author Contributions

Mengzhou Xue and Ruixue Wei conceived and designed the experiments. Yang Liu, Yun Chen, and Yuanyuan Liu performed the experiments. Yang Liu, Yun Chen, and Qingli Wang analyzed the data. Yang Liu, Yun Chen, and Zhe Li prepared all figures. Yang Liu, Yun Chen wrote the first draft of the manuscript. Mengzhou Xue, Ruixue Wei, and V. Wee Yong reviewed and edited. Yang Liu acquired funding, project administration, and supervision. All authors edited the manuscript and approved the final version of the manuscript.

## Funding

The work was supported by National Natural Science Foundation of China (82401559), Henan Province Key Research and Development and Promotion Program (232102311086), and The Health Commission of Henan Province (YQRC2023012, LHGJ20230327).

## Ethics Statement

All experiments involving animals were approved by the Ethics Review Committee of the Second Affiliated Hospital of Zhengzhou University.

## Conflicts of Interest

The authors declare no conflicts of interest.

## Supporting information


**Figure S1:** Biosafety assessment of MINO@PNM@CD147 in vivo.
HE staining of heart, liver, spleen, lung and kidney. (*n* = 3 per group).

**Figure S2:** The cell viability of HT‐22 cells after incubation with MINO and MINO@PNM@CD147 for 24 h (*n* = 3/group).

## Data Availability

The data that support the findings of this study are available on request from the corresponding author. The data are not publicly available due to privacy or ethical restrictions.
